# Checklist to assess Trustworthiness in RAndomised Controlled Trials (TRACT checklist): concept proposal and pilot

**DOI:** 10.1186/s41073-023-00130-8

**Published:** 2023-06-20

**Authors:** Ben W. Mol, Shimona Lai, Ayesha Rahim, Esmée M. Bordewijk, Rui Wang, Rik van Eekelen, Lyle C. Gurrin, Jim G. Thornton, Madelon van Wely, Wentao Li

**Affiliations:** 1 Department of Obstetrics and Gynaecology, Monash University, Clayton, Australia; 2 Aberdeen Centre for Women’s Health Research, University of Aberdeen, Aberdeen, UK; 3 Centre for Reproductive Medicine, Amsterdam UMC, Location AMC, Amsterdam, the Netherlands; 4 Department of Epidemiology & Data Science, Amsterdam UMC, Location VUmc, Amsterdam, the Netherlands; 5 Centre for Epidemiology and Biostatistics, School of Population and Global Health, The University of Melbourne, Parkville, Australia; 6 Faculty of Medicine & Health Sciences, University of Nottingham, Nottingham, UK; 7 Netherlands Satellite of the Cochrane Gynaecology and Fertility Group, Amsterdam UMC, Amsterdam, the Netherlands

**Keywords:** Research integrity, Trustworthiness, Randomised controlled trials, Checklist

## Abstract

**Objectives:**

To propose a checklist that can be used to assess trustworthiness of randomized controlled trials (RCTs).

**Design:**

A screening tool was developed using the four-stage approach proposed by Moher et al*.* This included defining the scope, reviewing the evidence base, suggesting a list of items from piloting, and holding a consensus meeting. The initial checklist was set-up by a core group who had been involved in the assessment of problematic RCTs for several years. We piloted this in a consensus panel of several stakeholders, including health professionals, reviewers, journal editors, policymakers, researchers, and evidence-synthesis specialists. Each member was asked to score three articles with the checklist and the results were then discussed in consensus meetings.

**Outcome:**

The Trustworthiness in RAndomised Clinical Trials (TRACT) checklist includes 19 items organised into seven domains that are applicable to every RCT: 1) Governance, 2) Author Group, 3) Plausibility of Intervention Usage, 4) Timeframe, 5) Drop-out Rates, 6) Baseline Characteristics, and 7) Outcomes.

Each item can be answered as either no concerns, some concerns/no information, or major concerns. If a study is assessed and found to have a majority of items rated at a major concern level, then editors, reviewers or evidence synthesizers should consider a more thorough investigation, including assessment of original individual participant data.

**Conclusions:**

The TRACT checklist is the first checklist developed specifically to detect trustworthiness issues in RCTs. It might help editors, publishers and researchers to screen for such issues in submitted or published RCTs in a transparent and replicable manner.

**Supplementary Information:**

The online version contains supplementary material available at 10.1186/s41073-023-00130-8.

## Introduction

The US Office of Research Integrity (ORI) defines research misconduct as “fabrication, falsification, or plagiarism in proposing, performing, or reviewing research, or in reporting research results. Research misconduct does not include unintended error or differences of opinion” [1].

The prevalence of researchers who have been involved in scientific misconduct is estimated to be around 2% [2] and, for randomized clinical trials (RCTs), this percentage is likely to be higher. Carlisle et al. reported recently that, based on assessment of review of individual participant data, 44% of submitted randomised controlled trials to the journal “Anaesthesia” were based on false data, with 26% of studies being entirely fabricated [3]. The corresponding estimates of false and fabricated trials run into the hundreds of thousands, and even a prevalence of ten or even hundred times lower would still point to thousands of “zombie trials” [4].

RCTs are recognized as the most reliable type of scientific investigation when assessing the effects of interventions. The results of RCTs are usually summarised in systematic reviews and meta-analyses, and subsequently used to support clinical recommendations in guidelines. This process is intended to improve the overall effectiveness and efficiency of medical interventions by providing trustworthy evidence [5, 6]. As RCTs are used to inform clinical guidelines, they directly influence patient care. If, however, some of these trials report a treatment effect that is based on false or fabricated data then it has the potential to adversely affect the health of patients. In fact, research misconduct in published RCTs has already been found to pollute the overall evidence base, with investigations from the UK indicating that results from retracted research can significantly affect results of meta-analyses or systematic reviews in which they are included, further contributing to potential adverse clinical impacts [7, 8]. Due to this influence, the trustworthiness of RCTs is potentially more important than that of other types of medical research.

RCTs have specific governance characteristics (including prospective trial registration requirements), detailed protocols (including documentation of study medication), and checklists that have to be completed at submission to journals [9, 10]. RCTs also have characteristics, absent in other forms of studies, that lend themselves to direct assessment of trustworthiness problems. For example, there should be comparable arms of trial participants and predictable patterns of differences in baseline variables between arms. In one of the most notorious cases of scientific misconduct, where more than 180 of anaesthetist Yoshitaka Fujii’s research papers have now been retracted, identification of statistical impossibility in the distribution of variables within his RCTs contributed in part to revealing this misconduct [11]. This highlights how deviations from the unique characteristics of RCTs may provide strong evidence of data manipulation within them.

In view of the importance of RCTs for clinical practice, and in light of their specific governance, methodological and submission requirements, there is both a need and an opportunity to develop tools that can be used to reliably assess, test and measure research integrity within and across submitted and already published RCTs whose trustworthiness has been questioned. During our efforts to assess the trustworthiness of RCTs we observed patterns, incorporated existing methods, and developed a pathway to assess potential problems [12–15]. Based on our experience and a subsequent systematic scoping review that assessed current methods of assessing research misconduct [16], we developed a prototype checklist to screen for trustworthiness issues due to possible scientific misconduct in RCTs [17]. This preliminary screening tool was then refined and developed into the version we present in this paper, the Trustworthiness in RAndomised Clinical Trials (TRACT) checklist. The objective of this study is to introduce this checklist screening tool as a way to assess the trustworthiness of and provide greater insight into integrity issues within RCTs. We plan for this tool to be widely used by reviewers and editors, as well as by those who perform systematic reviews, to help safeguard research integrity.

## Methods

Development of our screening tool was adapted from the four-stage approach proposed by Moher and colleagues: define the scope, review the evidence base, suggest a list of items from piloting, and hold a consensus process [18]. For the consensus process we held interactive meetings where stakeholders were asked to evaluate versions of the TRACT checklist and used questionnaires to help determine the final items to be included within.

### Defining the scope

We established a steering group of evidence-synthesis experts, with a background as clinical researchers, reviewers, editors, biostatisticians or evidence-synthesis specialists. The group was formed from colleagues who had been collaborating in the assessment of trials whose research integrity had been questioned, and also included journal editors and editors of specialist groups in Cochrane. Together this group agreed on the scope of the tool.

The primary decision of this steering group was to define the use of the screening tool. The screening tool would be developed as a quick checklist aiming to review articles and triage them according to their individual risk of research misconduct.

The steering group agreed to limit the screening tool to RCTs. The justification for this was that, while research misconduct is harmful for all types of research, RCTs are a crucial step in the assessment of medical interventions, prior to these interventions being applied in clinical practice and trustworthiness issues at this level stand to create direct harm for patients if their conclusions are not legitimate.

### Reviewing the evidence base

We conducted a scoping review of the literature for studies that mentioned a method for screening for or assessing and quantifying the extent of data integrity issues in health-related papers. The scoping review helped to provide an overview of all the current available methods to investigate research misconduct and identify the need for an effective and efficient checklist tool [16]. We subsequently conducted multiple systematic analyses (of which four have been published) to help inform the development of the screening checklist items and domains [12–15]. The findings and proposals from these systematic reviews and our experience led to the first version of the checklist.

### Piloting of the preliminary checklist

Over the last few years, we have assessed the integrity of over 300 RCTs in women’s health for a variety of reasons. We encouraged the use of the preliminary checklist across our research network as an informal and rough way to rapidly approximate the risk of research misconduct before embarking on more thorough checks. We received positive feedback from users – mostly junior academics and students from several countries – that in their assessments with this first version of the checklist it was able to successfully identify trustworthiness issues in RCTs that were later confirmed in more formal investigations led by journals or publishers. The utility of this preliminary checklist and informal feedback from users encouraged us to begin refining the checklist, and our group determined that directed feedback from stakeholders would assist in this process, making it more relevant to the research integrity objectives and more user friendly. EMB, WL, and BWM adjusted the draft of the checklists that was used for multiple rounds of piloting before presenting it to the consensus panel.

### Consensus process

We held an initial consensus meeting via videoconference with a panel with a range of stake-holders, including health professionals, reviewers, journal editors, policymakers, researchers and evidence-synthesis specialists. These stakeholders were invited to use the checklist draft to screen up to three articles and were then asked to complete a short questionnaire regarding their experience. For each checklist item, stakeholders were asked to rate both how useful it was in detecting possible breaches of data integrity and how easy it was to assess on a five-point Likert scale from ‘Not Useful/Easy’ to ‘Extremely Useful/Easy”. These selections were then allocated points (from 1 to 5) based on their rating (e.g. Not Useful = 1 and Extremely Useful = 5; Not Easy = 1 and Very Easy = 5). Therefore, a higher rating would mean more usefulness or easier for each item. The questionnaire also provided a free text section where stakeholders were also able to provide detailed feedback regarding their use of each domain as well as advise on any additional items they thought should be included in the checklist.

Based on their use of the checklist and the completion of the questionnaire, we compiled a feedback summary and calculated median aggregate scores for usefulness and ease. We regarded items with a median rating of 3 or more in both usefulness and ease as relevant and were eligible for inclusion in our final checklist version. We also organised for groups of three to four stakeholders to use the preliminary screening tool on the same set of three articles. Although they were not required to complete all three assessments, we compared responses where assessors had provided answers for the same article. When comparing the results there was an overall 65% agreement on item ratings based on the ‘Yes’ or ‘No’ answers in the preliminary checklist between users. Additionally, all item numbers except one had a 50% or more chance of similar results between users. The one exception was the ‘Effect size that is much larger than in other RCTs regarding the same topic’ in the Outcomes domain, and from reading feedback this was largely due to the article research field being unfamiliar to the user based on their research background. We discussed stakeholder feedback and adjusted the content, applicability and design of the screening checklist based on group consensus. On the basis of the agreed outcomes of the meeting, we adapted the draft and completed the final version of the checklist.

Ultimately all the items in the preliminary checklist were included in the final version, although additional comments were added or phrasing was changed to clarify particular items. Additionally, two items were added into the checklist – “Important prognostic factors are not reported as baseline characteristics” under the Baseline Characteristics domain and “Change in primary outcome from registration to publication” under the Outcomes domain.

## Rationale for included items

The TRACT checklist is sectioned into seven domains that are applicable to every RCT; governance, author group, plausibility of intervention usage, timeframe, drop-out rates, baseline characteristics, and outcomes. All of these domains combined provide an indication of the trustworthiness of the published RCT. These domains and the items included in them are discussed in further detail below.

### Governance

For each RCT, it is important to check registration of the study in an official trial registry (i.e. ICRTP Registry Network, clinicaltrials.gov). Registration should have occurred before the start of the study, and at least no later than 2 weeks after the start of participant recruitment. The absence of even retrospective registration is a cause for concern. One should look for mismatch in the planned number of participants in the trial registration, and the number participants actually recruited in the study. If there is a mismatch, there should be an explanation in the final report. Changes in the design and conduct of the trial registration should appear in the registration portal and can be tracked using online audit tools. The description of research ethics should be clear and preferably the ethics committee should be named, including a reference number of the ethics application. There should be a clear description of the process of informed consent as part of checking for ethical concerns with the study design.

### Author group

Aspects of author group may reflect the integrity of the study. RCTs with three or fewer authors, have a higher risk of trustworthiness issues, especially if they are multicentre RCTs [19]. Normally, larger multicentre studies would usually be conducted in collaboration with a clinical trials unit, and have (at least) statisticians, epidemiologists and medical scientists as co-authors. Retractions of other studies by one or more members of the author group may also arouse suspicion, especially if this retraction is not requested by the author(s). A search of the authors in Retraction Watch Database or PubMed can identify earlier retracted studies from an author or their institution. It is also important to note the rationale behind retractions and to identify if these have been made due to unintended errors that do not necessarily highlight research misconduct [20].

A large amount of RCTs published in a short timeframe by one trialist as first or last author or in one institute (e.g. > 3 per year as first author) should also be cause for concern. A search of the trial registers and databases of published papers and reports according to author names can provide an idea of the total number of studies that is performed during a timeframe. This is especially suspicious if an author is noted to be recruiting participants for their studies from a single institution; and on the other hand, concern may be somewhat mitigated if an author is performing trials at multiple different institutions or countries.

### Plausibility of intervention usage

The plausibility of a study can be assessed via its design. An example of implausible study design is the use of one placebo when there are two active interventions administered via different routes in a three-arm study. Studies that also describe their allocation concealment process either poorly (or not at all) should also raise concern as this detail provides validity and reliability to the study’s findings. Consider if the methods allow replication: whether the interventions and control/placebo are explained sufficiently enough to allow the process to be replicated in another experiment. In a similar vein, the description of the study design, and research methodology and the subsequent statistical analyses should be relevant and appropriate to the project being undertaken. In this domain, an understanding of clinical research methodology and biostatistics can aid in identifying trustworthiness issues.

### Timeframe

The study timeline is another important aspect to consider when appraising RCTs. The recruitment rate and the time between the end of recruitment to the submission of the manuscript have been identified by our study group as critical timeframes that require consideration, with timeframes that appear implausibly short being a point for concern.

In considering the speed of recruitment it is important to factor in the prevalence and incidence of the disease and the capacity of any recruiting centres, which usually can be obtained via an internet search. Two or more RCTs on the same topic executed simultaneously in one centre by the same author would, for example, be reason for concern. Additionally, the time needed to complete follow up is an important consideration for chronic diseases and interventions that require an extended time for assessing outcomes, such as assisted reproductive technology.

### Drop-out rates

Studies in which no participants were lost to follow up or for which no reasons are given for loss to follow up should be assessed with care, while taking into account the size of the study and the likely difficulty of follow up. There is often an expected level or rate of drop-out from RCT participants, especially when you consider long-term studies requiring prolonged and active engagement from participants that may prompt greater non-compliance (e.g. a questionnaire administered to participants at several points in time with large number of items); therefore lack of missing data points should be regarded as suspicious. On the other hand, a RCT study that has one point of data collection and a relatively simple technique to record of outcomes with no adequate explanation of drop-out, is similarly suspicious.

A similar drop-out rate for different treatments arms is often seen as a reassurance against bias; however, Bell et al*.* determined that equal number of drop-outs rates do not guarantee unbiased results in RCTs [21]. Still, some RCTs may fabricate their drop-out rates to appear more similar and less suspicious overall. Therefore, perfectly balanced drop-out rates in round numbers in particular (i.e. two groups of 50, two groups of 100, etc.) with no explanation for such balance should raise concern, especially if this implies that a larger group of participants were ultimately enrolled than were initially recruited.

### Baseline characteristics

Baseline characteristics provide important demographic and medical information on the target population of the RCT, and whether or not balance of baseline characteristics was achieved to the extent that would be expected from the randomised allocation of interventions. A lack of detail or the absence of baseline characteristics in a paper prevents the ability of others to assess whether groups apparently generated by randomisation have been appropriately balanced. Additionally, this domain may also reveal implausible patient characteristics when considering common sense, previously reported results, and local data (e.g. standard deviations are similar for completely different characteristics with different means or distributions). We also note that baseline characteristics in near-perfect balance is unrealistic if allocation was determined via randomised allocation of a sample of participants from a real population. Studies relating to specific research fields or medical conditions should highlight important and pertinent prognostic factors at baseline, and the absence of summary reporting of these should be flagged. Differences in baseline characteristics from different studies on the same topic, or differences in uniform characteristics such as body length are also reason for concern.

### Outcomes

When assessing study outcomes, effect sizes should be taken into consideration. If RCTs of the same topic with similar patient demographics indicating that effect size should be similar is instead substantially different, this should raise concern. Assessing the heterogeneity of studies is commonly used in meta-analyses and provides detail on the variability of included studies. Conflicting information between outcomes should also be assessed – for example, if a study has less clinical pregnancies (pregnancy confirmed via the presence of a gestation sac on ultrasound) compared to ongoing pregnancies (often defined as the presence of a viable pregnancy with cardiac activity noted from at least 12 weeks gestation), which should be impossible. It is also important to consider the primary outcome detailed at trial registration (if applicable) and check if this has now changed at publication [22].

## Using the checklist

The TRACT checklist (Additional file 1: Table S1) is designed to efficiently highlight the areas and as well as levels of concern within each domain via the item ratings – either ‘No Concerns’, ‘Some Concerns’/’Not Applicable’, or ‘Major Concerns’. The screening tool requires information found in the full text of the trial report and trial registration (if applicable). For each item, the user should choose one rating and address the rationale for the chosen rating in the ‘Support of Judgement’ section. There is also a free-text space for users to add additional comments about other integrity issues if required. After items are marked, a judgement can be made by user of the overall integrity risk level of the RCT being assessed, and from there further investigations could be undertaken. A non-exhaustive matrix of the particular risk levels for an item is given in Table 1, along with some guiding comments.

**Table 1:** A guiding matrix for using the TRACT Screening Checklist

*DOMAIN*	*ITEM*	*‘NO CONCERNS’ EXAMPLE*	*‘SOME CONCERNS’ EXAMPLE*	*‘MAJOR CONCERNS’ EXAMPLE*
Governance	Absent or retrospective registration of RCTs. This is relevant for RCTs commencing after 2010	Prospective registration	RCTs commencing before 2010	Absent or retrospective RCTs
	Discrepancy of > 15% between the intended sample size in the trial registration compared to the actual sample size achieved in the RCT *If registration is retrospective, then by definition the RCT sample size will be the same as the registered number*	Discrepancy of < 15%	No trial registration to provide intended sample size	Discrepancy of > 15%
	Absent or vague description of research ethics or apparent concerns regarding ethics	Thorough description of ethical concerns and research ethics obtained	Unclear ethical concerns in the setting of ethics approval	Absent description
Author Group *Consider using publisher services such as Scopus or Clarivate to identify authors and their publications*	Number of authors $\le$ 3 or low author to study size ratio	^a^		
	Other studies of authors have been retracted not on request of the authors *Consider checking *http://retractiondatabase.org/RetractionSearch.aspx	No identifiable retracted studies	Retracted studies on the request of the author with unclear explanation	Known retracted studies
	Large number of RCTs published in a short time frame by one author/in one institute *This ‘number’ is subjective based on the field of study, author or author group and timeframe*	^a^	Identical common names of authors with multiple articles in a short time frame	^a^
Plausibility of Intervention Usage	Insufficient or implausible description of allocation concealment (e.g. two interventions but only one placebo) *Consider if the interventions and control/placebo are explained sufficiently enough to be repeated in another experiment*	Description of allocation concealment is detailed enough to replicate	Allocation concealment described is plausible and reasonable but requires more detail	No description of allocation concealment
	Unnecessary or illogical description of methodological standards (e.g. use of sealed envelopes in a placebo-controlled trial)	Methodology ideal for study design	Methodology reasonable but not gold standard for study design	Methodology inappropriate for study design
Timeframe	Fast recruitment of participants within the study time (especially single centre studies)	^a^		
	Short or impossible time frame between ending recruitment/follow up and submission of the paper (take into account time to outcome e.g. live birth, pregnancy outcome etc.) *The recruitment time frame is from the date of the first recruited patient to the date of the last recruited patient*	^a^		
Drop-Out Rates	Zero participants lost to follow up or no reasons mentioned for loss of follow up *Especially in cases of long follow-up (e.g. multiple months) and/or multiple cycles of or long-lasting interventions*	Rationale for patients lost to follow-up provided	Patients lost to follow-up with insufficient rationale provided	Zero patients lost to follow-up despite long follow-up period
	Ideal number of losses to follow up resulting in perfectly rounded number in each group (e.g. groups of 50 or 100)	^a^	Almost exact numbers lost to follow-up group	Exact and -perfectly rounded numbers lost to follow-up in each group
Baseline Characteristics	No or few baseline (< 5) characteristics presented	An adequate amount of baseline characteristics that are relevant to the study	An adequate amount of baseline characteristics provided but with unclear relevance	No baseline characteristics provided
	Implausible patient characteristics judging from common sense, the literature and local data (e.g. similar standard deviations for completely different characteristics with different means and distributions)	^a^		
	Perfect balance for multiple baseline characteristics or significant/large differences between baseline characteristics	^a^		Identical numbers between groups of multiple baseline characteristics
	Important prognostic factors are not reported as baseline characteristics	^a^		
^a^Outcomes	Effect size that is much larger than in other RCTs regarding the same topic *Consider utilising meta-analyses if available*	^a^		
	Conflicting information between outcomes (e.g. more ongoing pregnancies than clinical pregnancies)	^a^		
	Change in primary outcome from registration to publication	No change in primary outcome	Single primary outcome in registration included as part of multiple primary outcomes in publication	Primary outcome in registration no longer mentioned or analysed in publication

^a^The rating for these items will need to be taken in the context of their research field

## Discussion

In this article, we have presented a screening checklist tool to detect publication trustworthiness issues in RCTs. This TRACT checklist is designed to screen papers and triage their risk of data fabrication to allow for better detection of research misconduct. Our checklist is straightforward, easy to apply, and enables the analysis of research misconduct without any significant prior experience or training. If a study is assessed and found to be suspicious, then reviewers should consider continuing with a more thorough investigation into the data integrity issues identified, in which we recommend assessment of the original data.

The TRACT checklist also has its limitations. Firstly, although we developed this checklist, it is not yet validated. Although the checklist was piloted and feedback was collected from a consensus meeting, a formalised Delphi method would allow for greater verification of the checklists’ utility and useability. Secondly, some patterns or reasons for concern are somewhat crude: other patterns of misconduct may not be picked up on by using this checklist. Fabricators can use the checklist to fabricate a paper that fits all the items of the checklist. As a screening tool, it may misclassify papers that either do or do not warrant further investigation. Lastly, using our checklist can be time consuming depending on the article being screened and the capacity and experience of users.

We have not provided a formal cut-off at which our checklist scores positive. This will be addressed in subsequent detection accuracy studies. Also, trustworthiness screening is an issue of common sense. Until now, the question ‘Are the data from a real study?’ and ‘Did the study actually take place “in real life”?’ have simply not been asked in assessment of submitted articles or during meta-analysis. Awareness of data-fabrication, even without using a formal score, will already make a large difference.

The TRACT checklist is the first checklist developed specifically to detect trustworthiness issues in RCTs. The need for such an assessment tool has previously been highlighted by our scoping review [16], and we believe the TRACT checklist can help editors, publishers and researchers who suspect scientific misconduct to make an efficient analysis of RCTs. If using this checklist raises suspicions or even provides evidence for research misconduct, the authors should be asked for an explanation. If they cannot provide a satisfactory or reasonable explanation, the next step for published articles is to consider investigation according to the Committee on Publication Ethics (COPE) guidelines. For researchers and readers who performed this assessment, they should contact the journals in which the papers are published. By highlighting the potential for research misconduct within RCTs, our TRACT checklist also aims to provide momentum and motivation for authors and journals to improve upon the trustworthiness of the RCTs that they publish.

We strongly believe that (anonymised) data sharing should become the standard before a paper can be accepted for publication, and that raw data should be publicly available at the moment of publication. Science is often thought of as a self-correcting system in which hypotheses and data are constantly being tested, replicated and validated, which is only possible when data is shared. The availability of participant data and the willingness to share these data may be a good indicator of quality, methodological soundness and integrity of RCTs [23, 24]. The burden of proof of integrity of a paper should be with the authors and not with the editors or peer reviewers.

Ultimately, the safety of patients was our primary concern and our motivation to develop this checklist. Providing medical treatment based on fraudulent research could be harmful, even if proposed treatment does nothing at all. Research misconduct is a major problem that all fields are facing, and the only way to tackle the problem is for all stakeholders to take shared responsibilities for improving the system and maintaining a high standard of research integrity, and for the publication and dissemination of the results of research that has been shown to meets these standards.

## Supplementary Information

**Additional file 1: Table S1.** The TRACT Screening Checklist.

## Data Availability

The data generated during the current study are available from the authors on reasonable request.
