# Supplementary material for: Checklist to assess Trustworthiness in RAndomised Controlled Trials (TRACT checklist): concept proposal and pilot
Source: Res Integr Peer Rev. 2023 Jun 20;8:6. doi: 10.1186/s41073-023-00130-8 (PMC10280869; doi:10.1186/s41073-023-00130-8)
Supplement: Supplementary file 1 — Additional file 1: Table S1. The TRACT Screening Checklist. [file 41073_2023_130_MOESM1_ESM.docx]

**Table S1: The TRACT Screening Checklist**

Overview

This screening tool aims to help identify and triage studies at risk of integrity issues. The checklist includes eight domains which are applicable to every RCT; governance, author group, plausibility of intervention usage, timeframe, drop-out rates, baseline characteristics and outcomes. The tool is a proposal to optimise our awareness of research integrity and has not yet been validated.

Users

This tool is designed to be used by clinical experts on articles in their field of study as a degree of clinical judgement and experience will be required for some items, and especially those using subjective or descriptive terms. It may also be beneficial to seek assistance from a statistician for some items.

Instructions for Use

The screening tool requires information found in the full text of the trial report and trial registration (if applicable) – please ensure you collect these prior to using the tool. Each item in the checklist is rated using a colour-coded system: green suggests ‘No Concerns’, yellow suggests ‘Some Concern/No Information’, and red suggests ‘Major Concern’. For each item, the user should choose one rating and address the rationale for the chosen rating in the ‘Support of Judgement’ section. There is also a free-text space for users to add additional comments about other integrity issues if required.

Details

| Article Title, Year |  |
| --- | --- |
| Author(s) |  |

Checklist

| *DOMAIN* | *ITEM* | *RATING* | | | | | *SUPPORT FOR JUDGEMENT* |
| --- | --- | --- | --- | --- | --- | --- | --- |
|  |  | No Concerns | Some Concerns/  No Information | | | Major Concerns |  |
| Governance | Absent or retrospective registration of RCTs. This is relevant for RCTs commencing after 2010 |  | |  | |  |  |
|  | Discrepancy of >15% between the intended sample size in the trial registration compared to the actual sample size achieved in the RCT |  | |  | |  |  |
|  | Absent or vague description of research ethics or apparent concerns regarding ethics |  | |  | |  |  |
| Author Group | Number of authors $\leq$3 or low author to study size ratio |  | |  | |  |  |
|  | Other studies of authors have been retracted not on request of the authors |  | |  | |  |  |
|  | Large number of RCTs published in a short time frame by one author/in one institute |  | | |  |  |  |
| Plausibility of Intervention Usage | Insufficient or implausible description of allocation concealment (e.g. two interventions but only one placebo) |  | | |  |  |  |
|  | Unnecessary or illogical description of methodological standards (e.g. use of sealed envelopes in a placebo-controlled trial) |  | | |  |  |  |
| Timeframe | Fast recruitment of participants within the study time (especially single centre studies) |  | | |  |  |  |
|  | Short or impossible time frame between ending recruitment/follow up and submission of the paper (take into account time to outcome e.g. live birth, pregnancy outcome etc.) |  | | |  |  |  |
| Drop-Out Rates | Zero participants lost to follow up or no reasons mentioned for loss of follow up |  | | |  |  |  |
|  | Ideal number of losses to follow up resulting in perfectly rounded number in each group (e.g. groups of 50 or 100) |  | | |  |  |  |
| Baseline Characteristics | No or few baseline (<5) characteristics presented |  | | |  |  |  |
|  | Implausible patient characteristics judging from common sense, the literature and local data (e.g. similar standard deviations for completely different characteristics with different means and distributions) |  | | |  |  |  |
|  | Perfect balance for multiple baseline characteristics or significant/large differences between baseline characteristics |  | | |  |  |  |
|  | Important prognostic factors are not reported as baseline characteristics |  | | |  |  |  |
| Outcomes | Effect size that is much larger than in other RCTs regarding the same topic |  | | |  |  |  |
|  | Conflicting information between outcomes (e.g. more ongoing pregnancies than clinical pregnancies) |  | | |  |  |  |
|  | Change in primary outcome from registration to publication |  | | |  |  |  |

Additional Comments

____________________________________________________________________________________________________________________________________________________________________________________________________________________________________________________________________________________________________________________________________________________________________________________________________________________________________________________________________________________________________________________
